# Treatment-refractory, juvenile-onset bipolar affective disorder

**DOI:** 10.4103/0019-5545.55962

**Published:** 2005

**Authors:** K. John Vijay Sagar

**Affiliations:** Assistant Professor

**Keywords:** Juvenile-onset bipolar affective disorder, attention-deficit hyperactivity disorder, treatment-refractory, clozapine

## Abstract

A case of juvenile-onset bipolar affective disorder with a childhood history of attention-deficit hyperactivity disorder (ADHD) is presented. As the patient was refractory to treatment with mood stabilizers, clozapine was given, which succeeded in achieving remission. The disorder's natural history needs further study. Data also need to be collected for optimal pharmacotherapeutic guidance.

## INTRODUCTION

An increasing amount of systematic research has challenged the traditional view that juvenile-onset bipolar disorder is a rare condition. The cumulative incidence of bipolar disorder in childhood and adolescence may equal the 1% rate in adults.^1^

In spite of the relatively recent conceptualization of the broad spectrum of bipolar disorder in adults, it is not clear whether the various components of this spectrum apply to the juvenile population as well. Thus, there is a need to explore and understand the natural history of bipolar disorder in all its forms in both children and adolescents.^2^

Although juvenile-onset bipolar disorder is becoming common, the optimal treatment approach for mania in this age group has not been adequately studied.

## THE CASE

An 18-year-old unmarried female, educated till class IV, hailing from middle socioeconomic status and an urban background with no family history, presented to us with a 2-week history of pervasive, elated mood with grandiosity, overdemanding attitude, talkativeness, increased psychomotor activity and anger outbursts.

The past history revealed that the patient had two similar episodes at the ages of 8 and 9 years. During these episodes, the patient had pervasive, elated mood, anger outbursts, increased psychomotor activity and reduced sleep. The first of these two episodes was precipitated by the death of her mother. The episodes lasted for 8 and 12 weeks, respectively. The patient received lithium 600 mg/day during these episodes. She was symptom-free in between the episodes and till she presented to us. No prophylactic mood stabilizer therapy was ever given to the patient

A diagnosis of bipolar affective disorder—current episode mania—was made after excluding drug use, thyroid dysfunction and other relevant medical conditions. From a review of the patient's chart it was found that at 5 years of age, she was seen by a child psychiatrist and diagnosed to have hyperkinetic conduct disorder (ICD-10). She had received haloperidol for 2 years and had complete remission of symptoms. A retrospective evaluation of the diagnosis made in childhood was done by parent interview with the Missouri Assessment of Genetics Interview in Children (MAGIC).^3^ It was concluded that the patient met the criteria for a diagnosis of attention-deficit hyperactivity disorder (ADHD) at the age of 5 years.

After ensuring that the haemogram, liver function tests and renal function tests were normal, the patient was started on lithium. As the patient failed to respond to an adequate dose of lithium, she was given an adequate trial of sodium valproate and carbamazepine monotherapy. As this therapeutic strategy failed to elicit a response, combinations of lithium and carbamazepine as well as that of lithium and valproate were tried. As the condition still remained treatment-refractory, clozapine was initiated at a dose of 12.5 mg/day and gradually titrated to 200 mg/day. The patient continued on clozapine and achieved complete remission by 4 months. Subsequently, she came for regular follow-up for 2 years and maintained remission. During follow-up, the patient was monitored for any adverse event with clozapine.

## DISCUSSION

This 18-year-old patient presented with three discrete episodes of mania. The first episode occurred at the age of 8 years qualifying it to be pre-pubertal-onset bipolar disorder. The first two episodes responded to mood stabilizer monotherapy (lithium). The patient had complete remission of symptoms and the interepisode interval was about 1 year. Before the present episode, the patient remained asymptomatic for about 9 years in spite of the absence of any prophylaxis. In view of the history of childhood ADHD, the cardinal diagnostic features of elated mood and grandiosity helped to clinch the diagnosis of mania.^4^

The occurrence of discrete, short-lived episodes of mania in this patient is in accordance with similar findings in Indian studies on juvenile-onset bipolar disorder.^5^,^6^ A high rate of recovery (defined as the absence of clinically significant mood symptoms for 8 weeks) from the index episode and a low rate of chronicity has been reported by some other Indian authors as well.^6^,^7^ This is in distinct contrast to the findings in western studies in which chronicity, psychosis, mixed features, atypical symptoms and a high incidence of rapid cycling were reported.^8^,^9^

In the context of a multimodal approach, the core treatment of early-onset bipolar affective disorder, though empirical, is pharmacological. Children with juvenile-onset bipolar disorder do not appear to respond well to mood stabilizers alone.^10^ Although mood stabilizers are the mainstay of treatment, there is increasing interest in the newer antipsychotics and anticonvulsants for the treatment of this disorder. Atypical antipsychotics have been considered in more severe and/or treatment-resistant manic or mixed episodes.^11^ Clozapine has been reported to be useful in treating adolescents either in conjunction with mood stabilizers or alone in treating refractory bipolar disorder.^12^,^13^ In the present case, clozapine monotherapy succeeded in achieving remission. The lack of response of the index manic episode to mood stabilizer therapy could be postulated to be due to the past history of childhood ADHD^13^ and prepubertal onset of the disorder.^14^

The natural history and management of juvenile bipolar disorder present more questions than answers. Treatment research in prepubertal bipolar disorder is in a rudimentary stage. More research is needed in all aspects of this disorder, especially various modes of treatment by controlled studies, and longitudinal course and diagnostic issues of the disorder.
